# Integrated Analysis of Oncogenic Networks in Colorectal Cancer Identifies GUCA2A as a Molecular Marker

**DOI:** 10.1155/2019/6469420

**Published:** 2019-07-28

**Authors:** Hui Zhang, Yuanyuan Du, Zhuo Wang, Rui Lou, Jianzhong Wu, Jifeng Feng

**Affiliations:** ^1^Research Center for Clinical Oncology, The Affiliated Cancer Hospital of Nanjing Medical University, Jiangsu Cancer Hospital, Jiangsu Institute of Cancer Research, Nanjing 210000, Jiangsu, China; ^2^Department of Oncology, The Affiliated Hospital of Jiangnan University, Wuxi 214000, Jiangsu, China

## Abstract

Colorectal cancer (CRC) is one of the most common and deadly malignancies in the world. In China, the morbidity rate of CRC has increased during the period 2000 to 2011. Biomarker detection for early CRC diagnosis can effectively reduce the mortality of patients with CRC. To explore the underlying mechanisms of effective biomarkers and identify more of them, we performed weighted correlation network analysis (WGCNA) on a GSE68468 dataset generated from 378 CRC tissue samples. We screened the gene set (module), which was significantly associated with CRC histology, and analyzed the hub genes. The key genes were identified by obtaining six colorectal raw data (i.e., GSE25070, GSE44076, GSE44861, GSE21510, GSE9348, and GSE21815) from the GEO database (https://www.ncbi.nlm.nih.gov/geo). The robust differentially expressed genes (DEGs) in all six datasets were calculated and obtained using the library “RobustRankAggreg” package in R 3.5.1. An integrated analysis of CRC based on the top 50 downregulated DEGs and hub genes in the red module from WGCNA was conducted, and the intersecting genes were screened. The Kaplan–Meier plot was further analyzed, and the genes associated with CRC prognosis based on patients from the TCGA database were determined. Finally, we validated the candidate gene in our clinical CRC specimens. We postulated that the candidate genes screened from the database and verified by our clinical pathological data may contribute to understanding the molecular mechanisms of tumorigenesis and may serve as potential biomarkers for CRC diagnosis and treatment.

## 1. Introduction

Colorectal cancer (CRC) is a malignant tumor that ranks third in terms of incidence and second in terms of mortality worldwide [1]. Similarly, the incidence and mortality of CRC rank fifth in China [2]. Despite dramatic reduction in the overall CRC incidence and mortality [3, 4], the morbidity rate in China is still rising from 2000 to 2011 [2]. Hence, further research is desperately needed to elucidate the causes for the increasing burden of CRC and to advance treatments for tumor subtypes with low response rates to current therapies. Treatment according to the distinctive tumor biology is an effective means to reduce the mortality of patients with CRC, as well as the detection of biomarkers that enable the stratification of patients with CRC into different prognostic subgroups and in relation therapeutic response [5]. Advances in RNA sequencing technologies and bioinformatics analysis provide novel potential biomarkers and drug targets for tumor treatment [6]. Weighted gene coexpression network analysis (WGCNA), as a systems biology algorithm, can enable the identification of highly coexpressed gene clusters (modules) [7]. Then, such interest modules and hub genes related to clinical traits can be screened out and used to identify candidate biomarkers [8]. The robust rank aggregation (RRA) method can be used to integrate multiple sets of chip data gene lists and to perform comprehensive reordering to find the most significant difference genes [9]. RRA prevents cross-platform standardization processing, and the number of samples per chip has no strict limit, which is of great significance for the effective evaluation of the results of different gene expression profiles [10].

In our study, we performed WGCNA on GSE68468 and screened out the key gene modules and hub genes significantly associated with the CRC histology. Additionally, we conducted RRA on six raw data (i.e., GSE25070, GSE44076, GSE44861, GSE21510, GSE9348, and GSE21815) and calculated the top 50 robust DEGs in all the six data by using the library “RobustRankAggreg” package. We analyzed the integrated genes between DEGs and hub genes in the key module by using Kaplan–Meier analysis in the TCGA database and obtained the candidate genes associated with OS. Finally, we validated the candidate gene in our clinical CRC specimens. The candidate genes screened from the database and verified using our clinical pathological data might have significant clinical implications for CRC diagnosis, treatment, and prognosis prediction.

## 2. Results

### 2.1. Weighted Gene Coexpression Network Construction and Module Detection

**Figure 1:**
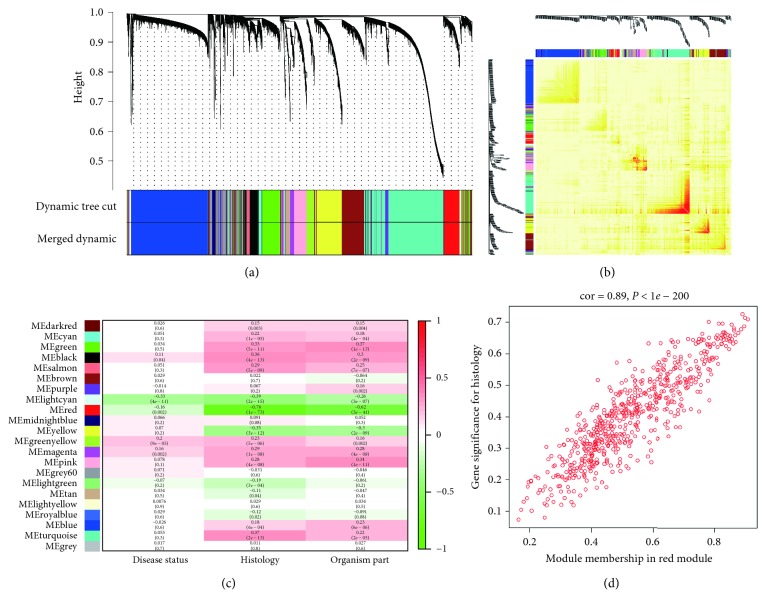
Modules defined by the WGCNA. (a) Dendrogram tree represents the clustering dendrogram of genes in GSE68468, and the bottom colorful bands represent 22 modules assigned by genes. (b) A network heatmap plot of 400 genes. The intensity of the red color indicates the strength of the correlation between pairs of modules on a linear scale. (c) Correlation between modules and clinicopathologic features. The correlation coefficient and corresponding *P* value of each module are shown in each cell. (d) Correlation (Pearson) between module membership and gene significance for the red module.

We performed WGCNA to find the highly correlated genes. The sample dendrogram and trait heatmap are shown in Figure S1A. As shown in Figure S1B, power value 4 was set to guarantee the high-scale independence and low mean connectivity of 13515 genes. We set the dissimilarity as 0.25 to merge similar modules (Figure S1C), and 22 modules were generated (Figure 1(a)). Furthermore, the interaction relationship network of 22 modules was plotted (Figure 1(b)). From those obtained modules, the red module had the deepest association with tumor histology (cor = −0.76, *P*=1*E* − 73), which was selected for further analysis (Figure 1(c)). Additionally, the module memberships in the red module and the gene significance had a high correlation (0.89) and a high *P* value (<1*e* − 200) (Figure 1(d)), suggesting its suitability for identifying the hub genes associated with CRC occurrence and metastasis.

### 2.2. Coexpression Network Construction and Hub Gene Identification

**Figure 2:**
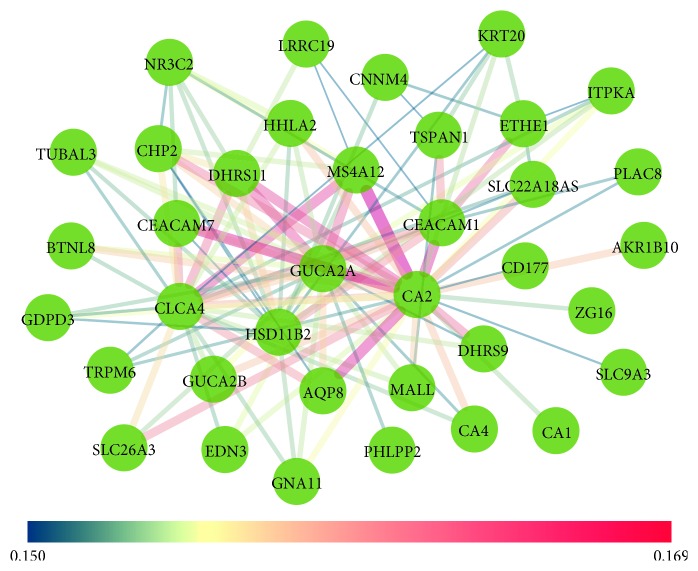
Coexpression network of hub genes. The hub genes in the red modules were filtered by a condition of the weight value (>0.15) and visualized using Cytoscape 3.6.0. The size and color of the edge represent the intensity of weight interaction in the network.

In our study, we obtained 616 genes in the red modules. The hub genes in the red module were filtered by a condition of the weight value being greater than 0.15, and a total of 37 edges were obtained and are visualized in Figure 2. The top 10 hub genes were CA2, MS4A12, DHRS11, GUCA2A, ETHE1, CLCA4, TSPAN1, HSD11B2, AQP8, and CHP2.

### 2.3. RRA Analysis

**Figure 3:**
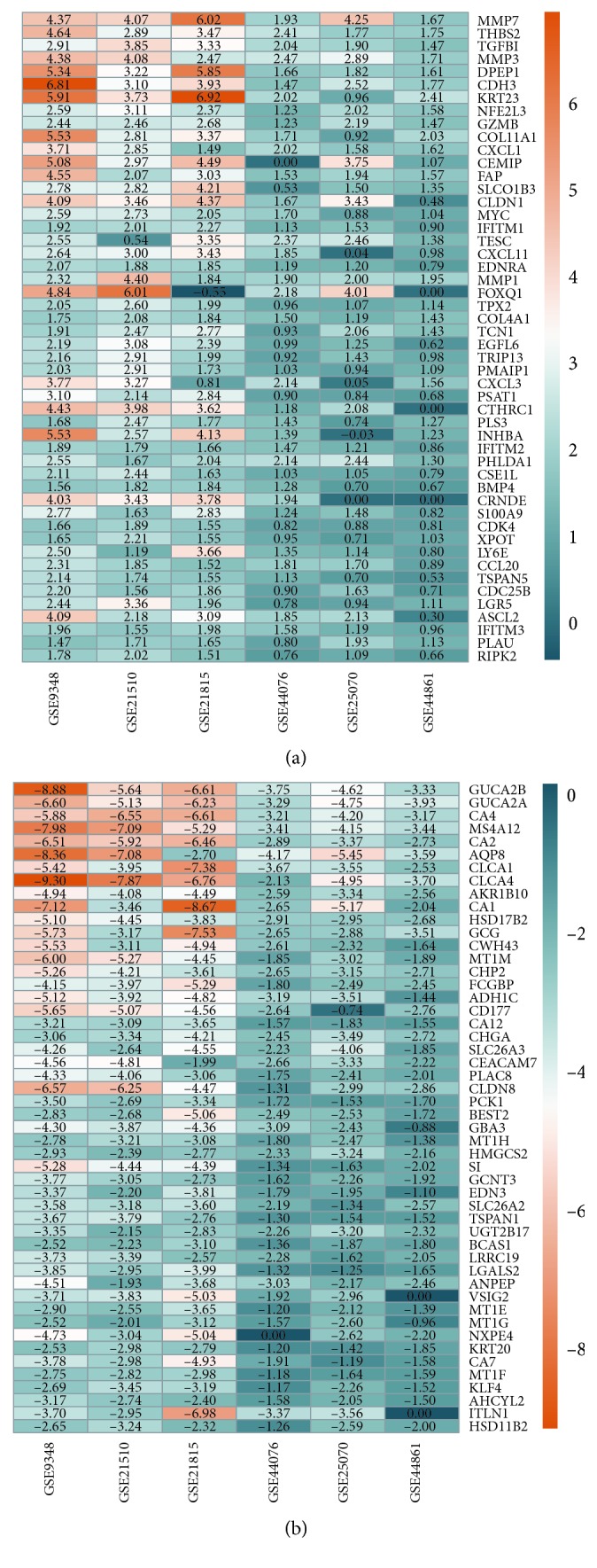
Top 50 robust DEGs identified in the six microarray data (i.e., GSE25070, GSE44076, GSE44861, GSE21510, GSE9348, and GSE21815) from the GEO database: (a) upregulated genes; (b)downregulated genes. These genes were ranked by adjusted *P* value.

We calculated DEGs expressed between the cancerous and adjacent tissues in each dataset and displayed the results by using volcano maps (Figures S2a–f). Then, DEGs in all six data were recalculated and reorganized using the library “RobustRankAggreg” package. A total of 464 robust DEGs were identified, including 176 upregulated genes and 288 downregulated genes (Table 1), by using adjusted *P*  *value* < 0.01 and |logFC| ≥ 1 as the cutoff criteria. The 50 most prominent up- and downregulated genes were screened and are visualized in Figures 3(a) and 3(b).

### 2.4. Survival Analysis in the TCGA Database and Validation in the GEO Database

**Figure 4:**
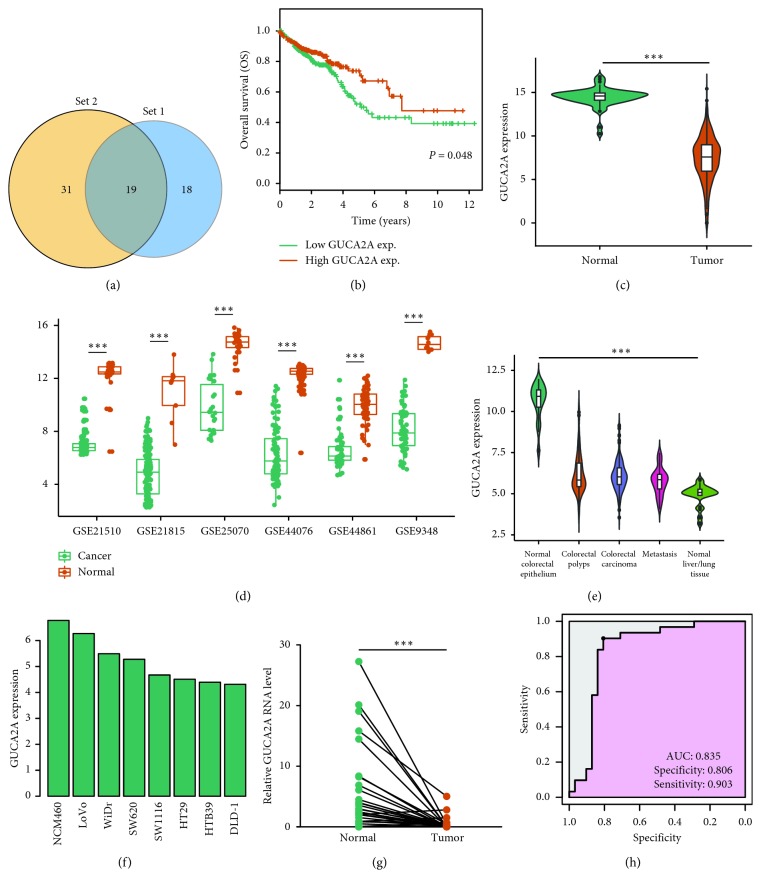
Aberrant expression of GUCA2A can be a candidate prognostic biomarker in CRC. (a) Venn diagram of the robust DEGs and genes in the red module revealed 19 interacting genes. Set 1: hub genes in the red module; Set 2: downregulated DEGs. (b) Five-year OS of CRC patients from the TCGA database (*P*=0.048). Patients with a low expression of GUCA2A have a significantly shorter OS. (c) GUCA2A expression in the TCGA database. (d) GUAC2A expression between CRC tissues and normal tissues in the six datasets. (e) GUAC2A expression in different tissue histology. (f) CRC cells in GSE68468. (g) GUCA2A expression levels in human CRC and their matched adjacent normal tissues. (h) ROC curves of tissue GUCA2A expression for differentiating CRC tissue from normal tissue. ^*∗∗∗*^ *P* < 0.001.

By taking the intersection of the 50 downregulated DEGs and hub genes in the red module from WGCNA (Figure 4(a)), we obtained 19 interacting genes.

K-M analysis was conducted to evaluate the relationship between gene expression and the overall survival (OS) of CRC, and only GUCA2A was clearly related to the OS of CRC patients in the TCGA database. Patients with a lower GUCA2A expression had a significantly shorter OS than those with a higher expression (*P* < 0.05) (Figure 4(b)). Obviously, GUCA2A was abnormally expressed in tumor tissues and was significantly different in TCGA and GEO databases (Figures 4(c) and 4(d)). We further validated the aberrant expression of GUCA2A in GSE68468, which contains both CRC tissues and cellular RNA-Seq data. Compared with adjacent normal tissues, the expression of GUCA2A in tumor and metastatic tissues was significantly low, and the normal liver and lung tissues had the lowest expression value (*P* < 0.05) (Figure 4(e)). The colonic epithelial cell (NCM460) has the highest GUCA2A expression compared with other CRC cells (Figure 4(f)).

### 2.5. Human Tissue Samples and Quantitative Real-Time PCR

We performed real-time PCR to examine the levels of GUCA2A in 31 paired CRC/adjacent tissues to further validate the dysregulated expression of GUCA2A (Figure 4(g)). Then, we further evaluated the diagnostic value of GUCA2A for CRC tissues and normal counterparts using ROC curve analyses. The results showed that the area under the ROC curve (AUC) was 0.835 (*P* < 0.001; sensitivity: 0.806; specificity: 0.903). These results suggest that GUCA2A downregulation may play an important role in colorectal tumorigenesis and has a potential diagnostic value for CRC patients.

### 2.6. Pathway Analysis

**Figure 5:**
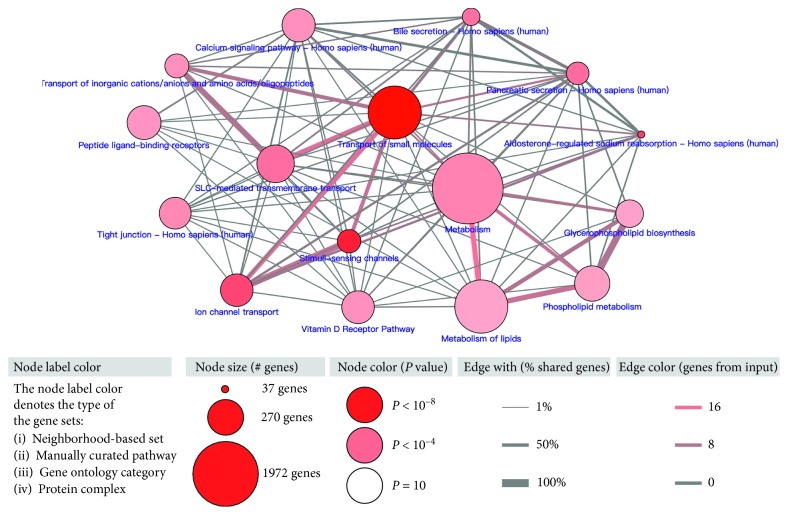
Pathway enrichment analysis for GUCA2A coexpressed genes.

The pathway enrichment analysis of GUCA2A coexpressed genes was conducted in the ConsensusPathDB human database. From the 16 pathways shown in Figure 5, the transport of small molecules and metabolism are prominent.

## 3. Discussion

Early detection and complete resection before metastasis are considered the only curative therapy for CRC [11]. The five-year survival rate of CRC patients was obviously better when the localized disease was detected at an early stage than that of CRC patients with distant metastasis. Cancer is a molecularly heterogeneous disease, and none of the currently identified biomarkers are sensitive and specific enough for reliable CRC screening in the clinical setting. Thus, identifying novel molecular biomarkers has significant clinical benefits.

In our study, we first performed WGCNA for GSE68468 and screened the pathologically related hub genes. We conducted RRA analysis for six datasets and screened the top 100 robust DEGs, which had a high or low expression in all expression profiles. By taking the intersection, we obtained 19 candidate genes, and only the expression of GUCA2A was associated with the OS of CRC patients in the TCGA database. We found that GUCA2A was prominent and top-ranking in both the hub gene network (Figure 2) and robust DEGs (Figure 3(b)), indicating its value in tumorigenesis.

Guanylin (GUCA2A) and uroguanylin (GUCA2B) are two peptide hormones that function as paracrine endogenous ligands for the guanylate cyclase-C (GUCY2C) receptor [12]. A previous study indicated the role of GUCY2C signaling in facilitating mucosal wounding and inflammation mediation, in part, through the control of resistin-like molecule *β* production [13]. GUCA2A, GUCA2B, and GUCY2C are downregulated in inflammatory bowel disease [14], which may have implications in inflammatory bowel disease pathogenesis. A recent study suggests that GUCY2C can suppress tumor progress [15] in the intestine, and the loss of the GUCY2C signaling cascade increases CRC susceptibility [16]. Intestinal homeostasis disruption and CRC tumorigenesis are associated with a fairly common loss of GUCA2A and GUCA2B [17–19]. Bashir et al. revealed the possibility of GUCA2A loss silencing GUCY2C, which leads to microsatellite instability tumor [20].

Consistent with the expression of GUCA2A in our study, the expression of GUCA2B was significantly downregulated in CRC tissues and had a relative high weight in the red module, which further indicates that GUCA2A and GUCA2B may play a consistent role in CRC neoplasia.

In conclusion, we revealed that GUCA2A was downregulated in CRC tissues. Aberrantly expressed GUCA2A can be a candidate marker of poor prognosis in patients with CRC, which may be a therapeutic target for precision medicine in cervical cancer. However, further studies are still needed to explore the underlying molecular mechanism through which GUCA2A plays a role in CRC. However, future in vivo and in vitro experiments are still required to explore the mechanisms underlying the roles of GUCA2A in CRC.

## 4. Methods

### 4.1. WGCNA Construction and Module Detection

We performed WGCNA to microarray data GSE68468 generated from 378 CRC tissue samples, and the “WGCNA” package in R 3.5.1 was used to construct a coexpression network. WGCNA seeks to identify modules of densely interconnected genes by searching for genes with similar patterns of connection strengths or high topological overlap. For each dataset, Pearson correlation coefficients were calculated for all pairwise comparisons of expressed genes across all samples. Genes with similar expression profiles were classified into modules based on the TOM dissimilarity with a minimum size of 30 for the gene dendrogram and visualized via hierarchical clustering [7]. Then, the modules whose eigengenes were highly correlated (correlation above 0.7) were merged. The gene network was visualized with randomly selected 400 genes.

The resulting Pearson correlation matrix was transformed into a matrix of connection strengths (that is, an adjacency matrix) by using a power function (connection). All the modules were assigned to the corresponding color. The relationships of modules and clinical traits (i.e., disease status, histology, and organism part) were calculated. Among these clinical traits, pathology, including normal mucosa, polyps, CRC tissue, CRC with metastases, and normal lung/liver tissues, can reflect the occurrence and metastasis of CRC. The associations of individual genes with the clinical trait, namely, gene significance (GS), and the module eigengenes, namely, module membership (MM), were evaluated. Then, the correlation between GS and MM was calculated, and the highly correlated interest module can be used to construct the coexpression network and identify the hub genes.

### 4.2. Coexpression Network Construction and Hub Gene Identification

The genes in the key modules screened from WGCNA were further analyzed.

We filtered the hub genes by a condition of the weight value (>0.15) and visualized them using Cytoscape 3.6.0 [21].

### 4.3. Robust Rank Aggregation (RRA) Analysis

To further increase the reliability of the results and screen out the ideal candidate, we enrolled six published colorectal microarray data (i.e., GSE25070, GSE44076, GSE44861, GSE21510, GSE9348, and GSE21815) [22–31] from the GEO database, which have 530 CRC tissues and 50 normal tissues in our study (Table 2). After screening DEGs (adjusted *P* value < 0.05 and |logFC| ≥ 2) in each dataset by using the “limma” package [32], the RRA method was used to identify significantly robust DEGs by using the library “RobustRankAggreg” package in R 3.5.1. The statistically significant DEGs were defined to have adjusted *P* value < 0.01 and |logFC| ≥ 1. Finally, the top 50 up- and downregulated DEGs were selected and visualized by using a heatmap.

### 4.4. Survival Analysis in the TCGA Database

In order to increase the reliability of the results, the intersection of hub genes in the red module from WGCNA and 50 downregulated robust DEGs was performed and analyzed. Kaplan–Meier (K-M) analysis was conducted to evaluate the relationship between gene expression and the overall survival (OS) of 617 CRC patients in The Cancer Genome Atlas (TCGA) database (https://cancergenome.nih.gov/). Patients were classified into high- or low-expression groups according to the median value. Then, genes associated with CRC survival were screened.

### 4.5. Human Tissue Samples and Quantitative Real-Time PCR

Overall, the 31 CRC and adjacent normal tissues obtained at Jiangsu Cancer Hospital (Nanjing, China) were frozen immediately after surgical resection and kept at 80°C until further analysis. Tumor histopathology was classified according to the World Health Organization Classification of Tumors system. The present study was done with the approval of the local ethics committee.

RNA isolation (Takara, Dalian, China) was performed according to the manufacturer's instructions. Reverse transcription was conducted with a PrimeScript RT reagent kit (Takara, Kusatsu City, Shiga, Japan) and the fluorescent quantitative experiment with ABI qPCR 7300 (Torrance, CA). The PCR reactant mix consisted of 2 *µ*l cDNA solution, 10 *μ*l 2× PowerUp™ SYBR™ Green Master Mix (Thermo Fisher Scientific, Carlsbad, CA), 0.5 *μ*l of 10 *μ*M forward and reverse PCR primer, and 7 *μ*l DNA template Nuclease-Free Water. The PCR conditions were set as follows: denaturation at 50°C for 2 min, 95°C for 2 min, 95°C for 15 s, and 60°C for 1 min with 40 cycles. A GAPDH primer set was used as an internal control.

### 4.6. Pathway Analysis

We performed pathway enrichment analysis for the coexpressed genes to explore the possible mechanism of the candidate gene in CRC. The coexpressed genes were obtained from the TCGA database, and the top 100 genes with the highest Pearson correlation coefficient were considered to be significantly coexpressed (Table 3). Pathway enrichment analysis was performed by using the ConsensusPathDB human database (http://cpdb.molgen.mpg.de/) [33]. The overrepresentation gene set analysis was utilized, and the following pathway databases were enrolled in our analysis: Manual upload, NetPath, SignaLink, PID, EHMN, HumanCyc, INOH, KEGG, BioCarta, WikiPathways, SMPDB, and PharmGKB. Minimum overlap input list >5 and *P* value cutoff <0.01 were considered significant enrichment.

## Figures and Tables

**Table 1:** Whole DEGs (176 upregulated genes and 288 downregulated genes) identified in the six microarray data from the GEO database by using adjusted *P* value < 0.01 and |logFC| ≥ 1 as the cutoff criteria.

Name	*P* value	Adjusted *P* value	LogFC
*Upregulated DEGs*			
MMP7	1.04*E* − 16	2.71*E* − 12	3.717319
THBS2	3.32*E* − 16	8.67*E* − 12	2.821997
TGFBI	7.96*E* − 16	2.08*E* − 11	2.584099
MMP3	1.59*E* − 15	4.15*E* − 11	2.998933
DPEP1	4.59*E* − 15	1.20*E* − 10	3.250292
CDH3	4.60*E* − 14	1.20*E* − 09	3.266416
KRT23	1.55*E* − 13	4.05*E* − 09	3.658825
NFE2L3	1.32*E* − 12	3.45*E* − 08	2.149137
GZMB	1.36*E* − 12	3.55*E* − 08	2.078558
COL11A1	3.42*E* − 12	8.95*E* − 08	2.728714
CXCL1	3.70*E* − 12	9.65*E* − 08	2.210194
CEMIP	4.96*E* − 12	1.30*E* − 07	2.894223
FAP	5.19*E* − 12	1.36*E* − 07	2.449763
SLCO1B3	6.55*E* − 12	1.71*E* − 07	2.197776
CLDN1	7.48*E* − 12	1.95*E* − 07	2.916603
MYC	1.15*E* − 11	2.99*E* − 07	1.831720
IFITM1	1.15*E* − 11	2.99*E* − 07	1.625712
TESC	2.53*E* − 11	6.61*E* − 07	2.108654
CXCL11	2.95*E* − 11	7.70*E* − 07	1.989137
EDNRA	3.02*E* − 11	7.89*E* − 07	1.496142
MMP1	3.29*E* − 11	8.60*E* − 07	2.399938
FOXQ1	5.40*E* − 11	1.41*E* − 06	2.749133
TPX2	5.42*E* − 11	1.41*E* − 06	1.634694
COL4A1	5.77*E* − 11	1.51*E* − 06	1.630033
TCN1	7.96*E* − 11	2.08*E* − 06	1.926601
EGFL6	9.09*E* − 11	2.38*E* − 06	1.752245
TRIP13	9.50*E* − 11	2.48*E* − 06	1.729510
PMAIP1	1.01*E* − 10	2.63*E* − 06	1.621771
CXCL3	1.19*E* − 10	3.10*E* − 06	1.933844
PSAT1	1.33*E* − 10	3.48*E* − 06	1.749995
CTHRC1	1.36*E* − 10	3.56*E* − 06	2.547903
PLS3	1.76*E* − 10	4.61*E* − 06	1.561012
INHBA	1.82*E* − 10	4.76*E* − 06	2.470231
IFITM2	2.22*E* − 10	5.81*E* − 06	1.477155
PHLDA1	2.77*E* − 10	7.25*E* − 06	2.025229
CSE1L	2.77*E* − 10	7.25*E* − 06	1.509054
BMP4	4.28*E* − 10	1.12*E* − 05	1.311002
CRNDE	4.93*E* − 10	1.29*E* − 05	2.196882
S100A9	5.05*E* − 10	1.32*E* − 05	1.793278
CDK4	6.20*E* − 10	1.62*E* − 05	1.268501
XPOT	6.54*E* − 10	1.71*E* − 05	1.350678
LY6E	6.54*E* − 10	1.71*E* − 05	1.773015
CCL20	8.45*E* − 10	2.21*E* − 05	1.681642
TSPAN5	8.63*E* − 10	2.25*E* − 05	1.300671
CDC25B	9.62*E* − 10	2.51*E* − 05	1.477785
LGR5	1.03*E* − 09	2.69*E* − 05	1.765688
ASCL2	1.06*E* − 09	2.78*E* − 05	2.270850
IFITM3	1.12*E* − 09	2.94*E* − 05	1.535829
PLAU	1.35*E* − 09	3.52*E* − 05	1.449998
RIPK2	1.40*E* − 09	3.65*E* − 05	1.304180
COL5A2	1.42*E* − 09	3.72*E* − 05	1.709418
CXCL10	1.45*E* − 09	3.78*E* − 05	1.832510
DUSP14	1.49*E* − 09	3.89*E* − 05	1.604280
TRIB3	1.52*E* − 09	3.97*E* − 05	2.009118
CKMT2	1.71*E* − 09	4.46*E* − 05	2.047320
CEL	1.77*E* − 09	4.62*E* − 05	1.921534
TGIF2	1.82*E* − 09	4.74*E* − 05	1.594622
HOMER1	1.89*E* − 09	4.94*E* − 05	1.595741
SRPX2	1.97*E* − 09	5.15*E* − 05	1.759897
SLC7A5	2.05*E* − 09	5.37*E* − 05	1.907805
UBD	2.06*E* − 09	5.38*E* − 05	1.848031
GPSM2	2.90*E* − 09	7.58*E* − 05	1.310352
AZGP1	3.52*E* − 09	9.20*E* − 05	2.058214
AURKA	3.81*E* − 09	9.96*E* − 05	1.248453
STC2	3.90*E* − 09	0.000102	1.838371
SULF1	3.90*E* − 09	0.000102	2.003268
ATAD2	4.09*E* − 09	0.000107	1.500415
TMEM158	4.11*E* − 09	0.000107	1.212075
PPAT	4.12*E* − 09	0.000108	1.305250
SPP1	4.13*E* − 09	0.000108	2.205887
CXCL2	4.36*E* − 09	0.000114	1.826375
COL1A2	4.44*E* − 09	0.000116	1.882105
SLC7A11	5.29*E* − 09	0.000138	1.208023
TIMP1	5.69*E* − 09	0.000149	1.661168
MMP12	6.08*E* − 09	0.000159	1.933314
SCD	6.11*E* − 09	0.000160	1.564105
SOX9	6.43*E* − 09	0.000168	1.352557
CXCL9	6.92*E* − 09	0.000181	1.461166
HSPH1	8.01*E* − 09	0.000209	1.238503
GDPD5	8.12*E* − 09	0.000212	1.308392
CXCL8	8.27*E* − 09	0.000216	2.588362
COL1A1	8.38*E* − 09	0.000219	1.769908
FABP6	8.52*E* − 09	0.000223	2.079844
PMEPA1	8.52*E* − 09	0.000223	1.333566
ANXA9	9.83*E* − 09	0.000257	1.608179
TDGF1P3	1.15*E* − 08	0.000301	1.841176
CEP55	1.22*E* − 08	0.000319	1.470764
COL10A1	1.27*E* − 08	0.000331	1.780470
MCM10	1.37*E* − 08	0.000358	1.211513
MPP6	1.51*E* − 08	0.000396	1.432320
PROX1	1.58*E* − 08	0.000413	1.364230
TEAD4	1.65*E* − 08	0.000431	1.447381
CLDN2	1.72*E* − 08	0.000449	1.698260
ODAM	1.79*E* − 08	0.000468	1.471032
MTHFD2	1.83*E* − 08	0.000478	1.111261
KRT6B	1.85*E* − 08	0.000484	1.485079
HILPDA	1.87*E* − 08	0.000488	1.488635
DACH1	2.14*E* − 08	0.000559	1.610398
ESM1	2.17*E* − 08	0.000568	1.708858
CYP4X1	2.22*E* − 08	0.000581	1.382114
GNG4	2.25*E* − 08	0.000588	1.178933
ARID3A	2.35*E* − 08	0.000613	1.275140
PPBP	2.38*E* − 08	0.000621	1.748166
EPHX4	2.42*E* − 08	0.000633	2.381989
LRP8	2.67*E* − 08	0.000697	1.522085
PTP4A3	2.67*E* − 08	0.000697	1.183443
SERPINE2	2.67*E* − 08	0.000697	1.154912
AGT	2.81*E* − 08	0.000734	1.405610
C2	2.84*E* − 08	0.000743	1.372973
RRM2	2.86*E* − 08	0.000748	1.196973
NEBL	3.49*E* − 08	0.000912	1.521795
RPP40	3.49*E* − 08	0.000912	1.285132
ARNTL2	3.77*E* − 08	0.000984	1.419157
AHCY	3.84*E* − 08	0.001004	1.237807
GTF2IRD1	3.95*E* − 08	0.001032	1.371062
WNT2	4.00*E* − 08	0.001044	1.452032
TOP2A	4.04*E* − 08	0.001057	1.372624
PUS7	4.09*E* − 08	0.001069	1.249772
NMU	4.30*E* − 08	0.001122	1.284463
COL6A3	4.44*E* − 08	0.001160	1.270615
RNF43	4.57*E* − 08	0.001195	1.397306
COL5A1	4.69*E* − 08	0.001226	1.191502
CDK1	4.70*E* − 08	0.001228	1.317782
ETV4	4.86*E* − 08	0.001271	2.204983
PTGS2	5.04*E* − 08	0.001317	1.233879
CHEK1	5.20*E* − 08	0.001358	1.126745
MMP10	5.41*E* − 08	0.001414	1.195256
FZD3	5.52*E* − 08	0.001442	1.089222
UBE2C	5.81*E* − 08	0.001518	1.241558
RNASEH2A	6.16*E* − 08	0.001609	1.071208
ENC1	6.27*E* − 08	0.001637	1.375197
IGF2BP3	6.37*E* − 08	0.001665	1.082776
VSNL1	6.75*E* − 08	0.001764	1.786437
GALNT6	6.79*E* − 08	0.001774	1.266153
ERP27	6.97*E* − 08	0.001821	1.218536
PROCR	7.73*E* − 08	0.002020	1.376152
PAFAH1B3	7.88*E* − 08	0.002058	1.100418
RFC3	8.14*E* − 08	0.002126	1.372173
NKRF	8.14*E* − 08	0.002127	1.043072
CXCL5	9.69*E* − 08	0.002531	1.294918
SQLE	1.03*E* − 07	0.002700	1.308167
EXOSC5	1.08*E* − 07	0.002828	1.132422
S100P	1.13*E* − 07	0.002944	1.551099
ELOVL5	1.27*E* − 07	0.003331	1.176539
ZAK	1.29*E* − 07	0.003360	1.541699
TCFL5	1.45*E* − 07	0.003786	1.270140
LAPTM4B	1.67*E* − 07	0.004376	1.026874
CEBPB	1.70*E* − 07	0.004431	1.333258
SLCO4A1	1.83*E* − 07	0.004790	1.606904
TTK	1.89*E* − 07	0.004934	1.386804
TMEM97	1.98*E* − 07	0.005179	1.082142
CHI3L1	2.01*E* − 07	0.005239	1.621308
PPM1H	2.10*E* − 07	0.005475	1.353835
SORD	2.14*E* − 07	0.005591	1.037586
CST1	2.20*E* − 07	0.005747	1.894531
CENPF	2.46*E* − 07	0.006422	1.024242
GTF3A	2.47*E* − 07	0.006451	1.215577
APCDD1	2.59*E* − 07	0.006772	1.580775
KIF4A	2.59*E* − 07	0.006772	1.267447
MFAP2	2.65*E* − 07	0.006914	1.397671
CCNB1	2.77*E* − 07	0.007241	1.203637
GDF15	2.88*E* − 07	0.007514	1.563948
NXT1	2.93*E* − 07	0.007667	1.046948
SHMT2	2.95*E* − 07	0.007697	1.002641
SNTB1	2.96*E* − 07	0.007726	1.291950
TACSTD2	2.97*E* − 07	0.007754	1.640872
SNX10	2.99*E* − 07	0.007814	1.021758
SLC12A2	3.04*E* − 07	0.007933	1.134681
TNFRSF12A	3.13*E* − 07	0.008190	1.332447
SLC5A6	3.25*E* − 07	0.008488	1.064032
RUVBL1	3.26*E* − 07	0.008513	1.066507
FXYD5	3.32*E* − 07	0.008680	1.172860
ZNF239	3.35*E* − 07	0.008745	1.158161
REG1A	3.40*E* − 07	0.008876	1.168719
OLFML2B	3.57*E* − 07	0.009326	1.332885
ERCC6L	3.63*E* − 07	0.009472	1.325393

*Downregulated DEGs*			
GUCA2B	4.94*E* − 21	1.29*E* − 16	−5.47325
GUCA2A	5.63*E* − 20	1.47*E* − 15	−4.98756
CA4	9.10*E* − 20	2.38*E* − 15	−4.93587
MS4A12	2.15*E* − 19	5.61*E* − 15	−5.22647
CA2	3.16*E* − 19	8.27*E* − 15	−4.64843
AQP8	7.18*E* − 19	1.88*E* − 14	−5.22336
CLCA1	1.67*E* − 17	4.37*E* − 13	−4.41598
CLCA4	2.30*E* − 17	6.01*E* − 13	−5.78604
AKR1B10	3.47*E* − 17	9.06*E* − 13	−3.66480
CA1	1.37*E* − 16	3.58*E* − 12	−4.85127
HSD17B2	1.42*E* − 15	3.72*E* − 11	−3.65299
GCG	1.86*E* − 15	4.87*E* − 11	−4.24507
CWH43	3.10*E* − 15	8.09*E* − 11	−3.35700
MT1M	6.63*E* − 15	1.73*E* − 10	−3.74527
CHP2	6.63*E* − 15	1.73*E* − 10	−3.59700
FCGBP	9.37*E* − 15	2.45*E* − 10	−3.35836
ADH1C	1.89*E* − 14	4.93*E* − 10	−3.66678
CD177	7.18*E* − 14	1.88*E* − 09	−3.56989
CA12	1.04*E* − 13	2.73*E* − 09	−2.48416
CHGA	1.04*E* − 13	2.73*E* − 09	−3.21194
SLC26A3	1.09*E* − 13	2.85*E* − 09	−3.26689
CEACAM7	1.16*E* − 13	3.02*E* − 09	−3.26188
PLAC8	1.75*E* − 13	4.58*E* − 09	−2.93747
CLDN8	1.79*E* − 13	4.67*E* − 09	−4.07661
PCK1	3.95*E* − 13	1.03*E* − 08	−2.41432
BEST2	5.80*E* − 13	1.52*E* − 08	−2.88517
GBA3	6.08*E* − 13	1.59*E* − 08	−3.15495
MT1H	7.81*E* − 13	2.04*E* − 08	−2.45079
HMGCS2	1.28*E* − 12	3.35*E* − 08	−2.63704
SI	1.53*E* − 12	3.99*E* − 08	−3.18335
GCNT3	1.86*E* − 12	4.87*E* − 08	−2.55705
EDN3	2.02*E* − 12	5.29*E* − 08	−2.36855
SLC26A2	2.58*E* − 12	6.74*E* − 08	−2.74311
TSPAN1	3.02*E* − 12	7.89*E* − 08	−2.43173
UGT2B17	3.26*E* − 12	8.51*E* − 08	−2.68448
BCAS1	3.43*E* − 12	8.96*E* − 08	−2.14870
LRRC19	4.61*E* − 12	1.20*E* − 07	−2.60514
LGALS2	5.96*E* − 12	1.56*E* − 07	−2.50281
ANPEP	8.52*E* − 12	2.23*E* − 07	−2.96251
VSIG2	8.52*E* − 12	2.23*E* − 07	−2.90829
MT1E	9.09*E* − 12	2.37*E* − 07	−2.30341
MT1G	9.49*E* − 12	2.48*E* − 07	−2.13170
NXPE4	9.66*E* − 12	2.53*E* − 07	−2.93788
KRT20	9.69*E* − 12	2.53*E* − 07	−2.12657
CA7	1.29*E* − 11	3.38*E* − 07	−2.72816
MT1F	1.32*E* − 11	3.45*E* − 07	−2.15911
KLF4	1.38*E* − 11	3.59*E* − 07	−2.38010
AHCYL2	1.43*E* − 11	3.74*E* − 07	−2.24169
ITLN1	1.74*E* − 11	4.55*E* − 07	−3.42798
HSD11B2	2.10*E* − 11	5.49*E* − 07	−2.34259
NR3C2	2.53*E* − 11	6.60*E* − 07	−2.37317
EPB41L3	2.57*E* − 11	6.72*E* − 07	−2.39867
ITM2C	2.67*E* − 11	6.97*E* − 07	−2.12884
MMP28	2.86*E* − 11	7.48*E* − 07	−1.93771
ZG16	3.09*E* − 11	8.06*E* − 07	−4.23120
ABCG2	3.75*E* − 11	9.80*E* − 07	−3.20141
SLC4A4	4.13*E* − 11	1.08*E* − 06	−3.28247
SCGB2A1	4.33*E* − 11	1.13*E* − 06	−2.48958
MALL	4.61*E* − 11	1.20*E* − 06	−2.29999
TUBAL3	4.92*E* − 11	1.29*E* − 06	−2.39086
RUNDC3B	5.33*E* − 11	1.39*E* − 06	−1.89913
NR1H4	5.50*E* − 11	1.44*E* − 06	−2.46055
SLC30A10	5.68*E* − 11	1.48*E* − 06	−3.22414
PIGR	6.05*E* − 11	1.58*E* − 06	−2.18018
MUC2	6.24*E* − 11	1.63*E* − 06	−2.62907
HPGD	6.74*E* − 11	1.76*E* − 06	−2.64497
TSPAN7	6.84*E* − 11	1.79*E* − 06	−2.30396
PAPSS2	1.30*E* − 10	3.39*E* − 06	−1.78856
SLC17A4	1.31*E* − 10	3.43*E* − 06	−2.31184
DHRS9	1.36*E* − 10	3.56*E* − 06	−2.74003
CAPN9	1.65*E* − 10	4.32*E* − 06	−1.77713
PTPRH	1.70*E* − 10	4.43*E* − 06	−1.93474
SST	1.81*E* − 10	4.73*E* − 06	−2.35448
HEPACAM2	1.82*E* − 10	4.76*E* − 06	−2.85009
SMPDL3A	2.03*E* − 10	5.31*E* − 06	−1.85516
UGT2A3	2.16*E* − 10	5.65*E* − 06	−2.42200
TRPM6	2.42*E* − 10	6.34*E* − 06	−2.40017
CES2	2.58*E* − 10	6.74*E* − 06	−1.80950
NR5A2	2.64*E* − 10	6.91*E* − 06	−1.65550
DHRS11	2.77*E* − 10	7.25*E* − 06	−2.40964
ENTPD5	2.88*E* − 10	7.52*E* − 06	−1.91140
PLCL2	2.98*E* − 10	7.79*E* − 06	−1.71267
MYO1A	2.98*E* − 10	7.79*E* − 06	−1.82841
BTNL8	3.28*E* − 10	8.58*E* − 06	−2.68136
PLCD1	3.40*E* − 10	8.88*E* − 06	−1.69423
ADTRP	3.71*E* − 10	9.69*E* − 06	−2.66899
ARL14	4.37*E* − 10	1.14*E* − 05	−2.10661
PDZD3	4.78*E* − 10	1.25*E* − 05	−1.73340
SCNN1B	5.25*E* − 10	1.37*E* − 05	−2.51853
MEP1A	5.25*E* − 10	1.37*E* − 05	−2.15953
C2orf88	5.40*E* − 10	1.41*E* − 05	−2.60606
GPA33	5.69*E* − 10	1.49*E* − 05	−1.85470
SCIN	6.20*E* − 10	1.62*E* − 05	−2.19971
MAOA	6.61*E* − 10	1.73*E* − 05	−1.79573
PIGZ	6.89*E* − 10	1.80*E* − 05	−1.67717
PYY	6.96*E* − 10	1.82*E* − 05	−2.53788
C4orf19	7.26*E* − 10	1.90*E* − 05	−1.57357
TNFRSF17	7.47*E* − 10	1.95*E* − 05	−2.36650
ADAMDEC1	7.47*E* − 10	1.95*E* − 05	−2.39500
TEX11	8.71*E* − 10	2.28*E* − 05	−2.24440
CDHR2	9.44*E* − 10	2.47*E* − 05	−1.71114
TMEM171	9.64*E* − 10	2.52*E* − 05	−2.11860
STMN2	1.28*E* − 09	3.36*E* − 05	−2.05581
PKIB	1.45*E* − 09	3.78*E* − 05	−2.98037
CLIC5	1.57*E* − 09	4.11*E* − 05	−1.55858
BMP2	1.60*E* − 09	4.19*E* − 05	−1.76798
RETSAT	1.63*E* − 09	4.26*E* − 05	−1.68389
PADI2	1.69*E* − 09	4.42*E* − 05	−2.10023
SLC9A2	1.80*E* − 09	4.70*E* − 05	−1.55732
CYP2C18	2.16*E* − 09	5.65*E* − 05	−1.71017
C1orf115	2.32*E* − 09	6.05*E* − 05	−1.74460
CEACAM1	2.42*E* − 09	6.31*E* − 05	−1.84011
PDE9A	2.44*E* − 09	6.37*E* − 05	−2.05665
SLCO2A1	2.58*E* − 09	6.75*E* − 05	−1.53024
BCAR3	2.63*E* − 09	6.87*E* − 05	−1.50716
METTL7A	2.65*E* − 09	6.92*E* − 05	−1.62515
DSC2	2.65*E* − 09	6.92*E* − 05	−1.44448
IL1R2	2.75*E* − 09	7.19*E* − 05	−2.16852
CCDC68	2.75*E* − 09	7.19*E* − 05	−1.89156
GDPD3	2.78*E* − 09	7.28*E* − 05	−2.08667
PDE6A	2.81*E* − 09	7.34*E* − 05	−1.93721
EPHX2	3.12*E* − 09	8.16*E* − 05	−1.49186
SPIB	3.35*E* − 09	8.76*E* − 05	−2.27898
ITPKA	3.41*E* − 09	8.91*E* − 05	−1.62863
SCUBE2	3.66*E* − 09	9.57*E* − 05	−1.51582
AMPD1	4.13*E* − 09	0.000108	−1.81548
SELENBP1	4.61*E* − 09	0.000120	−2.04099
TFCP2L1	5.10*E* − 09	0.000133	−1.16817
BTNL3	5.13*E* − 09	0.000134	−2.10512
VIPR1	5.45*E* − 09	0.000142	−1.57631
ZZEF1	5.53*E* − 09	0.000144	−1.27744
NAT2	5.61*E* − 09	0.000147	−1.61058
CDHR5	5.81*E* − 09	0.000152	−1.97788
PTGDR	6.44*E* − 09	0.000168	−2.08658
HHLA2	7.58*E* − 09	0.000198	−1.91952
PLCE1	7.60*E* − 09	0.000199	−1.63459
BEST4	7.87*E* − 09	0.000206	−2.37175
FUCA1	8.12*E* − 09	0.000212	−1.41919
FGFBP1	8.12*E* − 09	0.000212	−1.45128
MT1X	8.24*E* − 09	0.000215	−1.87572
SLC16A9	8.52*E* − 09	0.000223	−1.97426
SEMA6A	8.52*E* − 09	0.000223	−1.66469
FXYD3	8.58*E* − 09	0.000224	−1.39744
DEFB1	8.94*E* − 09	0.000233	−1.76340
ACADS	9.22*E* − 09	0.000241	−1.70299
UGDH	9.50*E* − 09	0.000248	−1.45862
HRASLS2	1.06*E* − 08	0.000276	−1.48365
CYP4F12	1.09*E* − 08	0.000284	−1.26493
JCHAIN	1.10*E* − 08	0.000288	−2.33454
ABHD3	1.13*E* − 08	0.000295	−1.54939
VWA5A	1.23*E* − 08	0.000321	−1.12976
LDHD	1.27*E* − 08	0.000332	−2.10259
XDH	1.28*E* − 08	0.000334	−1.53406
INSL5	1.29*E* − 08	0.000337	−2.42430
SPINK5	1.38*E* − 08	0.000359	−2.43812
VILL	1.50*E* − 08	0.000391	−1.41689
SQRDL	1.51*E* − 08	0.000396	−1.38225
FABP2	1.52*E* − 08	0.000398	−1.88374
LGALS4	1.61*E* − 08	0.000421	−1.42830
DNASE1L3	1.65*E* − 08	0.000431	−1.82008
SLC44A4	1.66*E* − 08	0.000434	−1.59136
GOLM1	1.68*E* − 08	0.000439	−1.20581
RNF186	1.72*E* − 08	0.000450	−1.21896
GDPD2	1.85*E* − 08	0.000484	−1.90186
SLC22A18AS	1.85*E* − 08	0.000484	−1.62665
MGLL	1.91*E* − 08	0.000499	−1.32867
B3GNT6	1.92*E* − 08	0.000501	−1.59112
IL6R	1.92*E* − 08	0.000502	−1.86011
RAPGEFL1	1.92*E* − 08	0.000502	−1.32000
TRPM4	1.94*E* − 08	0.000508	−1.34414
ETFDH	2.00*E* − 08	0.000522	−1.52397
TMPRSS2	2.05*E* − 08	0.000536	−1.39816
APOBR	2.08*E* − 08	0.000544	−1.69871
APPL2	2.11*E* − 08	0.000551	−1.46951
IQGAP2	2.17*E* − 08	0.000566	−1.55628
FAM83E	2.17*E* − 08	0.000568	−1.17487
ETHE1	2.29*E* − 08	0.000599	−1.61458
FLVCR2	2.39*E* − 08	0.000624	−1.01211
ALDH1A1	2.41*E* − 08	0.000631	−1.28928
DENND2A	2.51*E* − 08	0.000657	−1.32793
HRCT1	2.57*E* − 08	0.000671	−1.58787
ATP8B1	2.69*E* − 08	0.000703	−1.00964
A1CF	2.74*E* − 08	0.000715	−1.36877
ELOVL6	2.96*E* − 08	0.000773	−1.08274
C11orf86	3.02*E* − 08	0.000789	−2.11210
TMEM100	3.14*E* − 08	0.000821	−1.69604
ASAP3	3.27*E* − 08	0.000854	−1.44474
S100A14	3.29*E* − 08	0.000859	−1.21353
PHLPP2	3.42*E* − 08	0.000892	−1.45264
LIMA1	3.47*E* − 08	0.000907	−1.20588
FABP1	3.51*E* − 08	0.000916	−1.62316
FMO5	3.62*E* − 08	0.000947	−1.41152
LRMP	3.64*E* − 08	0.000950	−1.84646
UGP2	3.68*E* − 08	0.000961	−1.28750
ATP2A3	3.74*E* − 08	0.000978	−1.51001
SLC22A5	3.74*E* − 08	0.000978	−1.30616
FAM46C	3.78*E* − 08	0.000989	−1.41521
LXN	3.97*E* − 08	0.001037	−1.10935
CDKN2B	3.98*E* − 08	0.001039	−2.23550
HMOX1	3.99*E* − 08	0.001043	−1.23547
NEDD4L	4.03*E* − 08	0.001054	−1.23680
CLDN7	4.18*E* − 08	0.001093	−1.29905
PARM1	4.21*E* − 08	0.001099	−1.52095
HSD3B2	4.29*E* − 08	0.001120	−2.26639
KIAA0513	4.39*E* − 08	0.001146	−1.22322
ACADVL	4.50*E* − 08	0.001176	−1.23429
SYTL2	4.74*E* − 08	0.001238	−1.07640
SLC1A1	4.79*E* − 08	0.001251	−1.40176
CNTN3	4.81*E* − 08	0.001256	−2.13362
CNNM4	4.97*E* − 08	0.001300	−1.36807
CASP7	5.04*E* − 08	0.001317	−1.34691
GLTP	5.30*E* − 08	0.001385	−1.40471
PGM1	5.49*E* − 08	0.001435	−1.23037
ERN2	5.60*E* − 08	0.001464	−1.10843
NAAA	5.72*E* − 08	0.001494	−1.45647
GNA11	6.10*E* − 08	0.001593	−1.40416
CCL23	6.37*E* − 08	0.001665	−1.50079
STYK1	6.40*E* − 08	0.001673	−1.56848
CFD	6.56*E* − 08	0.001715	−1.80374
C15orf48	6.82*E* − 08	0.001783	−1.45026
OASL	6.89*E* − 08	0.001800	−1.13508
GPT	6.99*E* − 08	0.001827	−1.44289
CLDN23	7.12*E* − 08	0.001859	−1.93577
MB	7.62*E* − 08	0.001991	−1.42542
HIGD1A	7.65*E* − 08	0.002000	−1.40813
EMP1	8.37*E* − 08	0.002188	−1.46200
ADH1B	8.46*E* − 08	0.002211	−2.01430
LPAR1	8.49*E* − 08	0.002219	−1.26559
GPAT3	8.70*E* − 08	0.002272	−2.01808
CHGB	8.90*E* − 08	0.002324	−1.55043
P3H2	9.09*E* − 08	0.002376	−1.48288
PRKACB	9.28*E* − 08	0.002424	−1.46492
OSBPL1A	9.84*E* − 08	0.002572	−1.16940
SPINK4	1.04*E* − 07	0.002716	−1.69181
MT2A	1.04*E* − 07	0.002726	−1.55590
SULT1B1	1.07*E* − 07	0.002807	−1.66731
NAT1	1.08*E* − 07	0.002828	−1.17391
ADRA2A	1.11*E* − 07	0.002892	−1.54370
MEP1B	1.13*E* − 07	0.002960	−2.16734
LPCAT4	1.18*E* − 07	0.003077	−1.02702
PCSK7	1.18*E* − 07	0.003077	−1.00185
ST6GALNAC1	1.18*E* − 07	0.003088	−1.65355
SGK2	1.21*E* − 07	0.003160	−1.41158
GRAMD3	1.26*E* − 07	0.003287	−1.25518
RIOK3	1.27*E* − 07	0.003331	−1.21281
CITED2	1.29*E* − 07	0.003374	−1.11561
CXCL12	1.41*E* − 07	0.003684	−1.54414
ITM2A	1.45*E* − 07	0.003786	−1.47880
SEPP1	1.51*E* − 07	0.003956	−1.10723
KBTBD11	1.53*E* − 07	0.004006	−1.05544
RHOF	1.55*E* − 07	0.004040	−1.11757
GSTA1	1.57*E* − 07	0.004105	−1.34829
GALNT12	1.61*E* − 07	0.004214	−1.23449
BCHE	1.70*E* − 07	0.004446	−1.57410
HGD	1.78*E* − 07	0.004657	−1.24750
HOXD1	1.83*E* − 07	0.004793	−1.79058
FGL2	1.90*E* − 07	0.004977	−1.31339
ATP8A1	2.03*E* − 07	0.005307	−1.05015
HIST1H1C	2.06*E* − 07	0.005393	−1.25170
MYOT	2.10*E* − 07	0.005480	−1.72660
PBLD	2.11*E* − 07	0.005502	−1.58127
RBM47	2.15*E* − 07	0.005614	−1.08864
CES3	2.18*E* − 07	0.005700	−1.45856
CD1D	2.18*E* − 07	0.005704	−1.29631
FAM150B	2.19*E* − 07	0.005719	−1.50655
RARRES1	2.23*E* − 07	0.005819	−1.46770
LGALS9	2.24*E* − 07	0.005842	−1.01981
KLK1	2.27*E* − 07	0.005936	−1.29450
TOX	2.27*E* − 07	0.005942	−1.29132
ACVRL1	2.36*E* − 07	0.006175	−1.13083
CPT2	2.44*E* − 07	0.006372	−1.22566
SGK1	2.57*E* − 07	0.006718	−1.64587
ALPI	2.62*E* − 07	0.006834	−1.09455
SLC51B	2.65*E* − 07	0.006919	−2.27581
SLC20A1	2.71*E* − 07	0.007076	−1.03315
SCGN	2.74*E* − 07	0.007164	−1.86108
ASPA	2.75*E* − 07	0.007186	−1.45497
FAM107B	2.81*E* − 07	0.007337	−1.24898
AGR3	2.82*E* − 07	0.007358	−1.80558
HOXA5	2.85*E* − 07	0.007437	−1.09160
NPY1R	2.97*E* − 07	0.007754	−1.92966
TNFSF10	2.97*E* − 07	0.007754	−1.20956
FAM107A	3.18*E* − 07	0.008318	−1.40373
MUC4	3.26*E* − 07	0.008520	−1.83234
TST	3.31*E* − 07	0.008645	−1.30505
NXPE1	3.57*E* − 07	0.009326	−1.90526
SPON1	3.60*E* − 07	0.009400	−1.78810
ST6GALNAC6	3.78*E* − 07	0.009874	−1.79857

FC: fold change.

**Table 2:** Detailed information on the six microarrays from the GEO database.

Reference	Tissue	GEO	Platform	Normal	Tumor
Hinoue et al. [22]	CRC	GSE25070	GPL6883	51	51
Cordero et al. [23–26]	CRC	GSE44076	GPL13667	148	98
Ryan et al. [27]	CRC	GSE44861	GPL3921	55	56
Tsukamoto et al. [28]	CRC	GSE21510	GPL570	25	123
Hong et al. [29]	CRC	GSE9348	GPL570	12	70
Mimori et al. [30, 31]	CRC	GSE21815	GPL6480	9	132

**Table 3:** GUCA2A coexpressed genes in the TCGA database.

Genes	Correlation	*P* value
GUCA2A	1	0
TMIGD1	0.81034948	6.78*E* − 145
CA4	0.78988642	1.02*E* − 132
GUCA2B	0.78006796	2.39*E* − 127
SCNN1B	0.76922102	9.92*E* − 122
CDKN2B-AS1	0.75976020	4.48*E* − 117
LINC00974	0.72826962	5.10*E* − 103
CA1	0.72589778	4.85*E* − 102
OTOP2	0.72524636	8.98*E* − 102
B3GNT7	0.69874083	1.59*E* − 91
CA2	0.69872813	1.60*E* − 91
CLDN8	0.69162255	5.79*E* − 89
CLCA4	0.68724381	2.01*E* − 87
MS4A12	0.67084612	6.81*E* − 82
CEACAM7	0.65697702	1.74*E* − 77
BTNL3	0.64672606	2.24*E* − 74
SLC25A34	0.64457072	9.77*E* − 74
C11orf86	0.64075941	1.28*E* − 72
CLDN23	0.64014863	1.93*E* − 72
EDN3	0.62755226	7.21*E* − 69
RP11-35P15.1	0.62358770	8.90*E* − 68
B3GALT5-AS1	0.62060619	5.75*E* − 67
SULT1A2	0.61591517	1.04*E* − 65
PYY	0.61445537	2.53*E* − 65
ZG16	0.61434490	2.71*E* − 65
ALPI	0.61109844	1.93*E* − 64
SLC25A47P1	0.60742616	1.73*E* − 63
BTNL8	0.60619878	3.58*E* − 63
BEST4	0.59344211	5.70*E* − 60
CD177	0.59322686	6.44*E* − 60
ST6GALNAC6	0.59074429	2.60*E* − 59
CDHR5	0.58538873	5.07*E* − 58
TRPM6	0.57770739	3.26*E* − 56
SEPP1	0.57757017	3.51*E* − 56
KRTAP13-2	0.57186209	7.21*E* − 55
SLC4A4	0.56853673	4.08*E* − 54
RP11-203J24.9	0.55828248	7.58*E* − 52
MYPN	0.55589062	2.50*E* − 51
RP11-202A13.1	0.54993651	4.66*E* − 50
SLC30A10	0.54640858	2.57*E* − 49
SLC26A3	0.54279335	1.45*E* − 48
SMPD1	0.54142083	2.77*E* − 48
CDKN2B-AS	0.53902904	8.55*E* − 48
RN7SKP127	0.53769807	1.59*E* − 47
HHLA2	0.53644205	2.86*E* − 47
TMEM82	0.53029307	4.85*E* − 46
LYPD8	0.52880179	9.56*E* − 46
CD177P1	0.52733551	1.85*E* − 45
SCNN1G	0.52521670	4.81*E* − 45
ABCC13	0.52358277	9.98*E* − 45
GDPD2	0.51942104	6.29*E* − 44
TTC22	0.51932599	6.56*E* − 44
B3GALT5	0.51777531	1.29*E* − 43
C2orf88	0.51755217	1.43*E* − 43
DHRS9	0.51539770	3.64*E* − 43
TSPAN1	0.51536862	3.69*E* − 43
INSL5	0.51280701	1.11*E* − 42
TMEM253	0.50785220	9.21*E* − 42
SDCBP2	0.50717257	1.23*E* − 41
SLC17A4	0.50515764	2.86*E* − 41
PLAC8	0.50131456	1.42*E* − 40
MMP28	0.49830422	4.90*E* − 40
PPY	0.49796769	5.62*E* − 40
PLA2G10	0.49555028	1.51*E* − 39
GLRA4	0.49493081	1.94*E* − 39
ITM2C	0.49231037	5.57*E* − 39
TRIM40	0.49190614	6.55*E* − 39
SMPDL3A	0.49188755	6.60*E* − 39
MALL	0.49120642	8.68*E* − 39
BCAS1	0.49019232	1.30*E* − 38
CDKN2B	0.48925853	1.89*E* − 38
SLC51B	0.48708976	4.46*E* − 38
BMP3	0.48536366	8.79*E* − 38
AMPD1	0.48202823	3.23*E* − 37
ENTPD5	0.47054777	2.56*E* − 35
PTPRH	0.46882533	4.87*E* − 35
RPL12P14	0.46759668	7.68*E* − 35
RP11-92A5.2	0.46652136	1.14*E* − 34
AC106869.2	0.46640139	1.19*E* − 34
MUC12	0.46578524	1.50*E* − 34
CASC18	0.46570335	1.55*E* − 34
PEX26	0.46071845	9.52*E* − 34
TMEM72	0.46026123	1.12*E* − 33
GLDN	0.45995677	1.25*E* − 33
TEX11	0.45994953	1.26*E* − 33
NAAA	0.45741196	3.13*E* − 33
TRANK1	0.45665719	4.10*E* − 33
SMIM6	0.45572985	5.71*E* − 33
LINC00507	0.44872641	6.70*E* − 32
AOC1	0.44821442	8.01*E* − 32
CDHR2	0.44663931	1.38*E* − 31
APPL2	0.44091022	9.81*E* − 31
TMPRSS2	0.43862005	2.12*E* − 30
CCDC152	0.43855903	2.17*E* − 30
TRPV3	0.43605403	5.02*E* − 30
NPY2R	0.43603750	5.04*E* − 30
GNA11	0.43389107	1.03*E* − 29
ACVRL1	0.43021941	3.44*E* − 29
SEMA6D	0.42947338	4.39*E* − 29
GPR15	0.42871749	5.62*E* − 29
RP1-117B12.4	0.40855340	3.19*E* − 26

## Data Availability

The data used to support the findings of this study are available from the corresponding author upon request.

## Supplementary Materials

Supplementary Materials S1: sample dendrogram and soft-thresholding value estimation. (A) Sample dendrogram and trait heatmap. The three traits correspond to the disease status, histology, and organism part, respectively. (B) Scale independence and mean connectivity of soft-thresholding values (*β*). (C) Clustering of module eigengenes. The dissimilarity was set as 0.25 to merge the similar modules. S2: volcano plots of DEGs between cancerous and adjacent tissues in six microarray data.
