# Supplementary material for: Integrated Analysis of Oncogenic Networks in Colorectal Cancer Identifies GUCA2A as a Molecular Marker
Source: Biochem Res Int. 2019 Jul 28;2019:6469420. doi: 10.1155/2019/6469420 (PMC6701329; doi:10.1155/2019/6469420)
Supplement: Supplementary Materials — S1: sample dendrogram and soft-thresholding value estimation. (A) Sample dendrogram and trait heatmap. The three traits correspond to the disease status, histology, and organism part, respectively. (B) Scale independence and mean connectivity of soft-thresholding values (β). (C) Clustering of module eigengenes. The dissimilarity was set as 0.25 to merge the similar modules. S2: volcano plots of DEGs between cancerous and adjacent tissues in six microarray data. [file 6469420.f1.pdf]

Figure s1

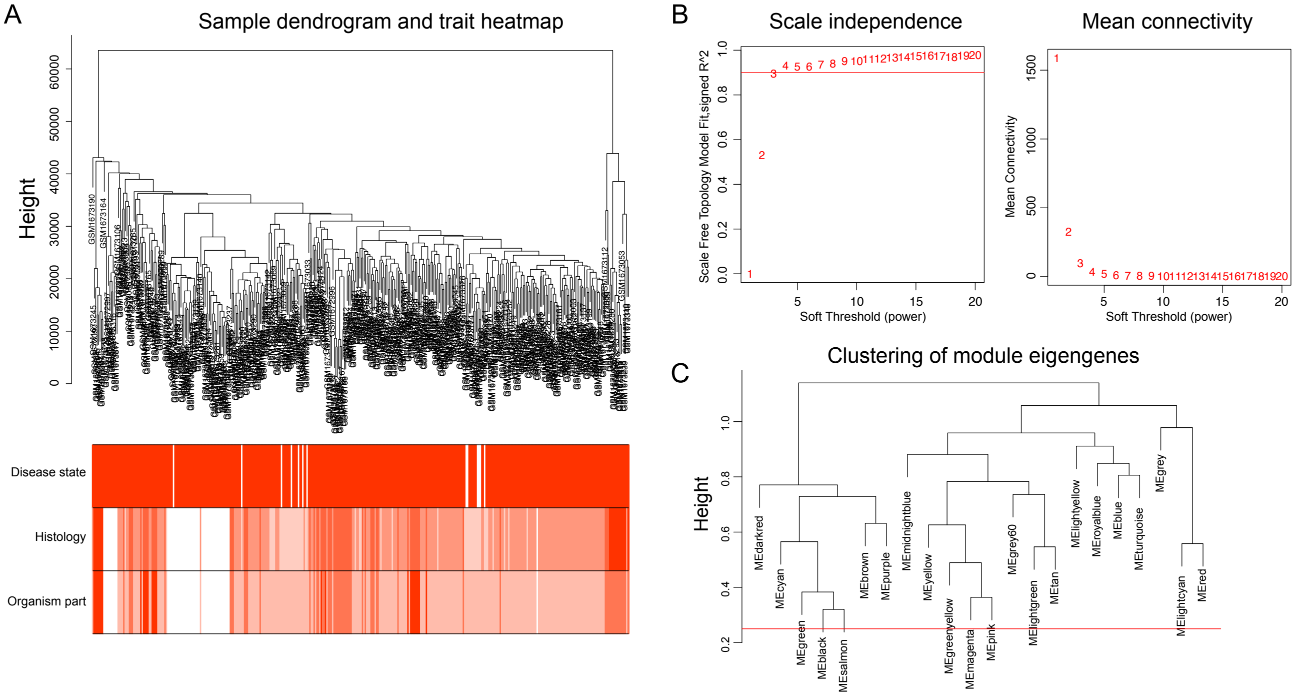

A
Sample dendrogram and trait heatmap
Height
0
10000
20000
30000
40000
50000
60000
Disease state
Histology
Organism part
B
Scale independence
Scale Free Topology Model Fit,signed R^2
Soft Threshold (power)
Mean connectivity
Mean Connectivity
Soft Threshold (power)
C
Clustering of module eigengenes
Height
MEdarkred
MEcyan
MEgreen
MEblack
MEsalmon
MEbrown
MEpurple
MEmidnightblue
MEyellow
MEgreenyellow
MEmagenta
MEpink
MEgrey60
MElightgreen
MEtan
MElightyellow
MEroyalblue
MEblue
MEturquoise
MEgrey
MElightcyan
MEred

Figure s2

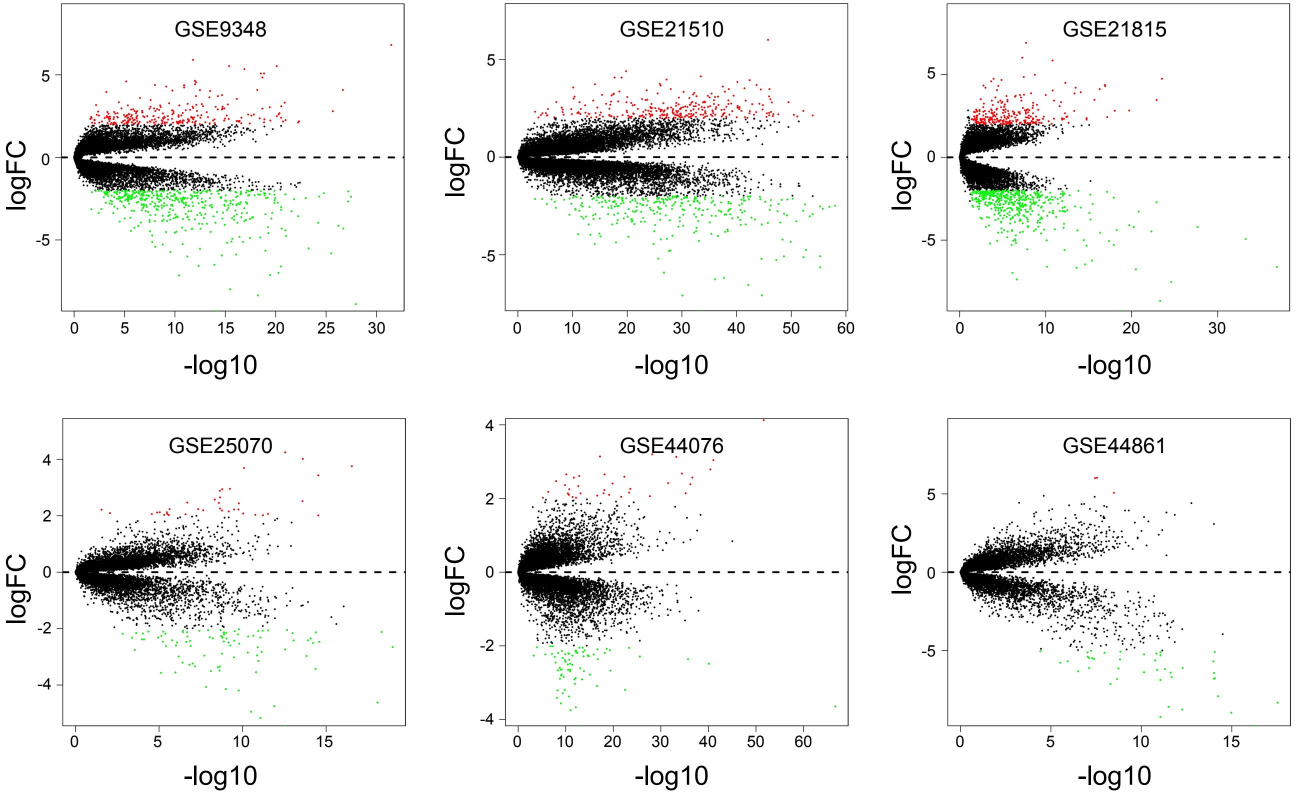
GSE9348
GSE21510
GSE21815
GSE25070
GSE44076
GSE44861
logFC
-log10
